# Changes in implant stability using different site preparation techniques: Osseodensification drills versus piezoelectric surgery. A multi‐center prospective randomized controlled clinical trial

**DOI:** 10.1111/cid.13140

**Published:** 2022-10-03

**Authors:** Claudio Stacchi, Giuseppe Troiano, Graziano Montaruli, Marco Mozzati, Luca Lamazza, Alessandro Antonelli, Amerigo Giudice, Teresa Lombardi

**Affiliations:** ^1^ Department of Medical, Surgical and Health Sciences University of Trieste Trieste Italy; ^2^ Department of Clinical and Experimental Medicine University of Foggia Foggia Italy; ^3^ Private Practice Turin Italy; ^4^ Department of Oral and Maxillofacial Sciences Sapienza University of Rome Rome Italy; ^5^ Department of Health Sciences Magna Græcia University Catanzaro Italy

**Keywords:** implant site preparation, implant stability, osseodensification, piezosurgery, resonance frequency analysis

## Abstract

**Introduction:**

Implant stability is influenced by bone density, implant design, and site preparation characteristics. Piezoelectric implant site preparation (PISP) has been demonstrated to improve secondary stability compared with conventional drilling techniques. Osseodensification drills (OD) have been recently introduced to enhance both bone density and implant secondary stability. The objective of the present multi‐center prospective randomized controlled trial was to monitor implant stability changes over the first 90 days of healing after implant bed preparation with OD or PISP.

**Methods:**

Each patient received two identical, adjacent or contralateral implants in the posterior maxilla. Following randomization, test sites were prepared with OD and control sites with PISP. Resonance frequency analysis was performed immediately after implant placement and after 7, 14, 21, 28, 60, and 90 days. Implants were then restored with single screw‐retained metal‐ceramic crowns and followed for 12 months after loading.

**Results:**

Twenty‐seven patients (15 males and 12 females; mean age 63.0 ± 11.8 years) were included in final analysis. Each patient received two identical implants in the posterior maxilla (total = 54 implants). After 1 year of loading, 53 implants were satisfactorily in function (one failure in test group 28 days after placement). Mean peak insertion torque (40.7 ± 12.3 Ncm and 39.5 ± 10.2 Ncm in test and control group, respectively) and mean implant stability quotient (ISQ) value at baseline (71.3 ± 6.9 and 69.3 ± 7.6 in test and control group, respectively) showed no significant differences between the two groups. After an initial slight stability decrease, a shift to increasing ISQ values occurred after 14 days in control group and after 21 days in test group, but with no significant differences in ISQ values between the two groups during the first 90 days of healing.

**Conclusion:**

No significant differences in either primary or secondary stability or implant survival rate after 1 year of loading were demonstrated between implants inserted into sites prepared with OD and PISP.

**What is known**

- Implant stability tends to decrease in the first weeks after implant placement due to peri‐implant bone remodeling following surgical trauma.
- Piezoelectric implant site preparation reduces stability decrease and favors earlier shifting from a decreasing to an increasing stability pattern, compared with conventional drilling.
- Osseodensification is a recently introduced, non‐subtractive implant site preparation technique, aiming to increase both primary implant stability and percentage of bone at the implant surface, compared with conventional drilling.

**What this study adds**

- No significant differences in primary or secondary stability were demonstrated between implants inserted into sites prepared with osseodensification drills and sites prepared with piezoelectric surgery.

## 1 INTRODUCTION

The early stage of bone repair response after dental implant insertion is a complex phenomenon in which the combined action of inflammatory cascade and immune system regulate new bone formation and neo‐angiogenesis.^1^ Among various factors influencing the healing process, excessive implant micro‐movement may compromise osseointegration by producing large interfacial strains which, especially in low‐quality bone, induce bone resorption and determine fibrous encapsulation of the fixture.^2^, ^3^, ^4^ Therefore, one of the main goals in implant surgery is the achievement of adequate primary stability, which is strictly related to both bone quality and quantity, implant design, and implant site preparation characteristics.^5^ Implant bed preparation should be performed with minimal trauma to the bone, avoiding overheating, and excessive compression of the cortical layer to prevent an excessive inflammatory phase potentially causing massive bone resorption, delayed healing or implant failure.^6^, ^7^, ^8^

The progressive drilling technique has always been the conventional approach to implant osteotomy, using increasing‐diameter twist drills rotating clockwise from 600 to 2000 rpm under copious irrigation.

Piezoelectric implant site preparation (PISP) has been proposed as an alternative technique to improve surgical control, safety, and bone healing response. Piezoelectric devices for bone surgery exploit ultrasonic vibrations of specific tips with three main features: (1) micrometric cutting with easy operative control, (2) selective cutting action on hard tissues, and (3) enhanced surgical visibility due to the cavitation effect of cooling saline solution.^9^, ^10^, ^11^ Moreover, PISP seems to improve healing response resulting in a limited stability decrease during the first weeks after implant placement and in an earlier shifting from a decreasing to an increasing stability pattern, compared with conventional drilling.^12^, ^13^, ^14^

Osseodensification drills (OD) is a recently introduced implant site preparation technique based on specially designed drills with large negative rake angles which, rotating counterclockwise, work as non‐cutting edges to expand the implant site and compact bone at the osteotomy walls.^15^ This non‐subtractive approach aims to increase primary stability and maintain secondary stability of dental implants inserted into low‐density bone compared with conventional drilling procedures.^16^, ^17^ Osseodensification protocols could help to obtain higher bone‐to‐implant contact and higher bone volume around implants.^18^

However, most studies analyzing OD were conducted in vitro, ex vivo or on an animal model. Well‐designed clinical trials on human subjects are necessary to fully elucidate the potential of this novel technique as an alternative to conventional implant site preparation in daily clinical practice. Therefore, the objective of the present randomized clinical trial was to compare stability changes of implants inserted into sites prepared using OD with implants inserted into sites prepared with PISP during the first 90 days of healing.

## 2 MATERIALS AND METHODS

### 2.1 Study design

The present study was a multi‐center, randomized controlled clinical trial with simple randomization (1:1 allocation ratio), conducted by six experienced operators, who enrolled and treated patients from June 2020 to February 2021. The present trial was reported following CONsolidated Standards of Reporting Trials guidelines. The study protocol was designed in accordance with recommendations expressed in the Fortaleza revision (2013) of the Helsinki Declaration for investigations on human subjects. The study protocol was approved by the relevant ethical committee (Comitato Etico Regione Calabria—Sezione Area Centro n. 418/2020) and retrospectively recorded in a public registry of clinical trials (https://clinicaltrials.gov—NCT05410405). A calibration meeting was held among all the clinical centers prior to the study to discuss and standardize operative protocols. Each clinician received written instructions regarding collection of experimental parameters in order to obtain acceptable inter‐examiner consistency. All patients, after being thoroughly informed about the study protocol, the treatment plan with its alternatives and any potential risk related to the therapy, signed a written informed consent to participate in the study and authorized the use of their data for research purposes.

The present superiority trial tested the null hypothesis of no difference in primary stability between implants inserted into sites prepared with different devices (OD [test group] and piezoelectric surgery [control group]), against the alternative hypothesis of a difference.

### 2.2 Patient selection

All partially edentulous patients needing two adjacent or contralateral implants in pristine bone in the maxillary premolar area were screened at the clinical centers for potential participation in this trial.

General inclusion criteria were the following: (I) age >18 years; (II) good general health; (III) absence of systemic disease affecting bone metabolism and wound healing; (IV) no regular medication consumption for at least 3 months prior to treatment; (V) patient willingness and capability to fully comply with the study protocol; (VI) signed written informed consent.

Local inclusion criteria were the following: (I) bone crest with a minimum of 6 mm width and 9 mm height above the maxillary sinus floor, with no concomitant or previous bone augmentation procedures; (II) healed bone crest (at least 6 months elapsed from tooth loss/extraction); (III) presence of opposing dentition.

Exclusion criteria were: (I) absolute medical contraindications to implant surgery^19^; (II) uncontrolled diabetes (HBA1c > 7.5%); (III) treated or under treatment with antiresorptives; (IV) irradiated in the head and/or neck area in the last 5 years; (V) patient pregnancy or lactating at any time during the study; (VI) poor oral hygiene and motivation (full mouth plaque score FMPS >25%); (VII) untreated periodontal disease; (VIII) psychiatric problems; (IX) alcohol or drug abuse.

All patients received oral hygiene instruction and professional deplaquing 1 week prior to implant surgery.

### 2.3 Surgical procedure

After raising a minimally invasive full‐thickness flap under local anesthesia (articaine 4% with epinephrine 1:100000), an independent assessor opened the randomization sealed opaque envelope, and the assigned treatment was revealed to the surgeon. Test and control sites were prepared with the same final diameter for insertion of two identical implants (4.1 × 8 mm or 4.1 × 10 mm Volution, i‐Res) during the same intervention. Selected implants present double‐threaded conical shape, moderately rough surface treatment, platform‐switched internal connection and machined implant neck.

**FIGURE 1:**
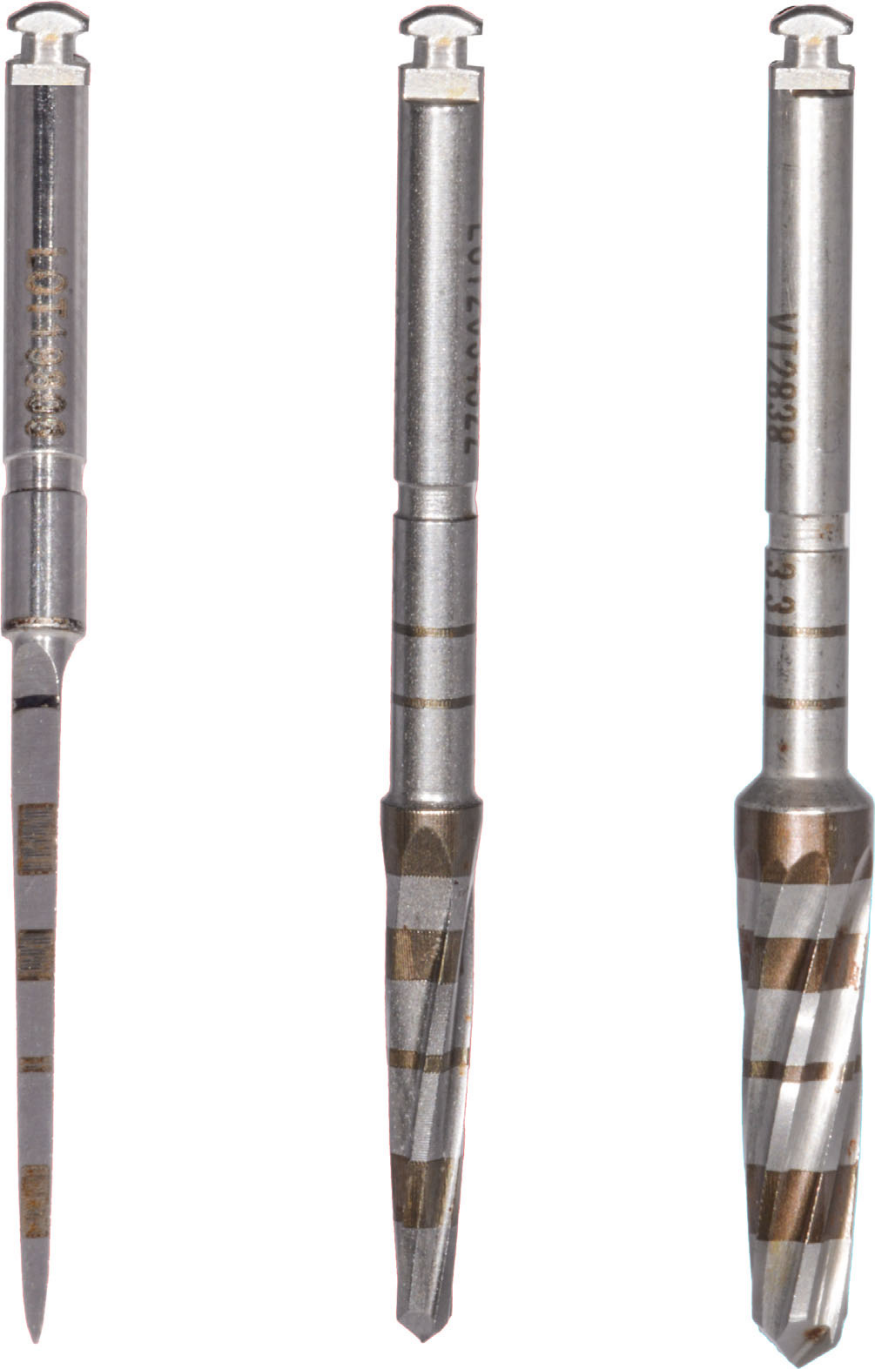
Osseodensification drills (Densah, Versah) were used at 1200 rpm in the following sequence: from left to right (I) pilot (clockwise); (II) WT1828 (counterclockwise), and (III) WT2838 (counterclockwise).

**FIGURE 2:**
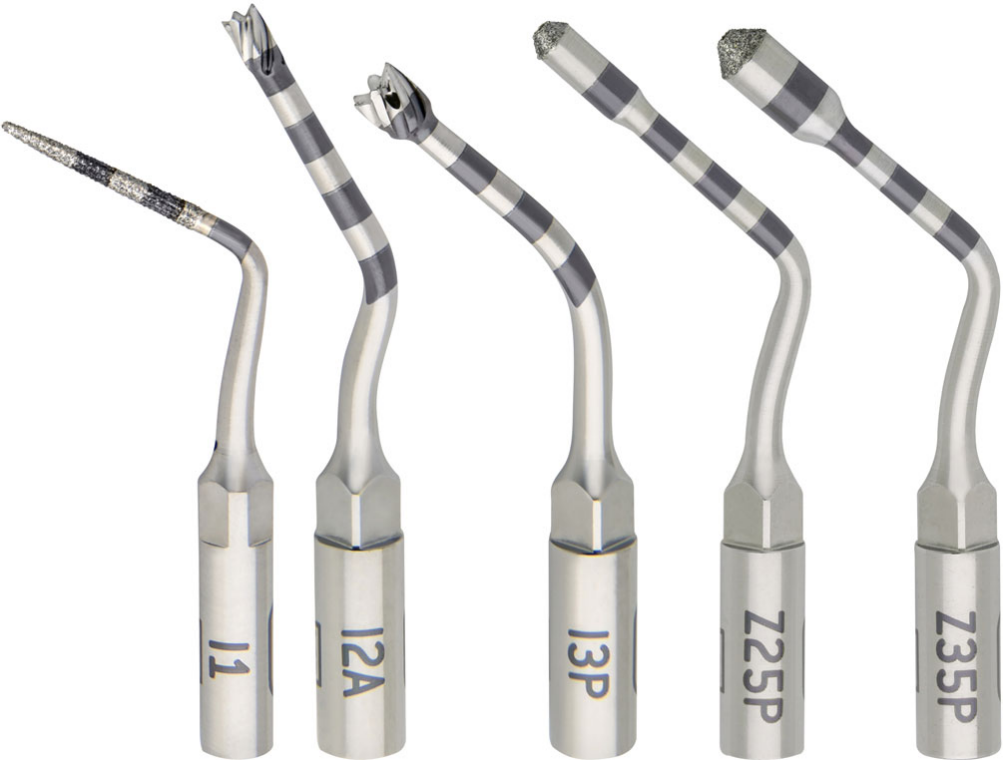
Piezoelectric tips (Piezomed, W&H) were used in the following sequence: from left to right (I) I1; (II) I2P; (III) Z25P; (IV) I3P, and (V) Z35P.

Test sites were prepared using osseodensification burs (Densah, Versah) at 1200 rpm in the following sequence: (I) pilot (clockwise); (II) WT1828 (counterclockwise), and (III) WT2838 (counterclockwise). These burs have a cutting chisel edge, a tapered shank and non‐cutting edges with four or more lands with a negative rake angle (Figure 1). Control sites were prepared using piezoelectric tips (Piezomed, W&H) in the following sequence: I1, I2P, Z25P, I3P, Z35P. These tips are diamond‐coated (I1; Z25P; Z35P) or smooth (I2P; I3P), and oscillate at 22–35 kHz with automatic setting of the right frequency by the surgical device (Figure 2). Insertion torque (Ncm) was recorded by the surgical motor (Implantmed, W&H) and implants were connected to 3 mm high, straight, multi‐unit abutments. A blinded operator measured implant stability at abutment level in mesio‐distal and bucco‐palatal directions using resonance frequency analysis (SmartPeg #A3, Osstell Beacon, Osstell) and implant stability quotient (ISQ) data were uploaded to a dedicated cloud‐based platform (Osstell Connect, Osstell). Flaps were sutured around multi‐unit abutment healing caps using the Sentineri technique^20^ and single stitches for unsubmerged healing using synthetic monofilament (Supramid 5/0, Butterfly Italia). Patients were prescribed antibiotics for 6 days (amoxicillin 1 g twice a day) and paracetamol 500 mg when needed.

Sutures were removed 7 days after surgery. A blinded assessor measured ISQ following the previously described protocol at 7, 14, 21, 28, 60, and 90 days. Implants were evaluated at every visit for mobility, pain and signs of infection. At 4 months, implants were restored with screw‐retained single metal‐ceramic crowns and followed up for at least for 12 months after prosthetic loading.

### 2.4 Predictor and outcome variables

The primary predictor variable was implant site preparation technique (OD vs PISP).

Primary outcome measure:

- implant primary stability (insertion torque and ISQ).

Secondary outcome measures:

- implant secondary stability pattern during the first 90 days after implant placement (ISQ);
- implant survival after 1 year of prosthetic loading;
- any complication or adverse event.

### 2.5 Sample size and randomization

Sample size calculation was performed by means of a web‐based software (https://app.sampsize.org.uk). As no previous studies comparing implant stability after OD or PISP are present in the literature, expected differences for sample size calculation were extrapolated from a recent randomized clinical trial comparing implant stability after OD or conventional drilling.^16^ A sample of seven patients from each group was required to detect significant differences (confidence level 5% with a statistical power of 90%), with an expected difference in implant primary stability of 19.0 ± 8.4 Ncm.

An investigator (GT), not involved in selection or treatment of patients, arranged a computer‐generated table using a balanced, randomly permuted block approach (www.random.org) to assign the two implants of each patient to the different groups (test and control). Programmed implant sites assigned to test group had the implant bed prepared with OD, while implant sites assigned to control group had the osteotomy prepared with piezoelectric surgery. Randomization codes were enclosed in numbered, identical, sealed, opaque envelopes. Envelopes were opened after flap elevation. Treatment allocation was concealed to the two operators in charge of enrolling and treating the patients in this trial.

### 2.6 Statistical analysis

An independent investigator (GT) performed data analysis using STATA 16.0 software (StataCorp). Implant stability was described at each single time point with a single ISQ value (mean of mesio‐distal and bucco‐palatal measurements).

The Shapiro–Wilk test was performed to assess data normality. Intra‐group differences were longitudinally analyzed using ANOVA and Scheffé tests, while inter‐group comparisons were assessed using the two‐sample Wilcoxon rank‐sum test. The level of significance was set at a *p*‐value lower than 0.05.

## 3 RESULTS

Forty‐two consecutive patients were screened for eligibility and, after applying inclusion and exclusion criteria, 27 patients (15 males and 12 females; age range 45–92 years, mean 63.0 ± 11.8 years; 8 smokers, 19 non‐smokers) were included in the present study. Each patient received two identical implants in the posterior maxilla (total = 54 implants); 13 patients received two 8 mm‐long implants and 14 patients received two 10 mm‐long implants (Table 1). Surgeries were performed by six experienced operators (CS, *n* = 5 patients; GM, *n* = 5 patients; MM, *n* = 5 patients; LL, *n* = 3 patients; AG, *n* = 4 patients; TL, *n* = 5 patients). No drop‐outs were recorded during the entire study period.

**TABLE 1:** Baseline characteristics of the sample

Gender	Male: 15 (55.6%)	Female: 12 (44.4%)
Age	63.0 ± 11.8 years—range 45–92 years	
Smoking status	19 (70.4%) non‐smokers	8 (29.6%) smokers
Implant length	8 mm (13 patients—48.1%)	10 mm (14 patients—51.9%)

Ninety days after insertion, 53 out of 54 implants resulted osseointegrated and were referred to the prosthodontist for subsequent rehabilitation (one failure was recorded in test group after 28 days). Two‐sample Wilcoxon rank‐sum test showed no significant differences (*p* = 0.82) between the two groups.

Except for the failed implant, no other local or systemic complications or adverse events were recorded at any site throughout the entire period of observation. At the last follow‐up (12 months of prosthetic loading), 53 implants were satisfactorily in function.

**FIGURE 3:**
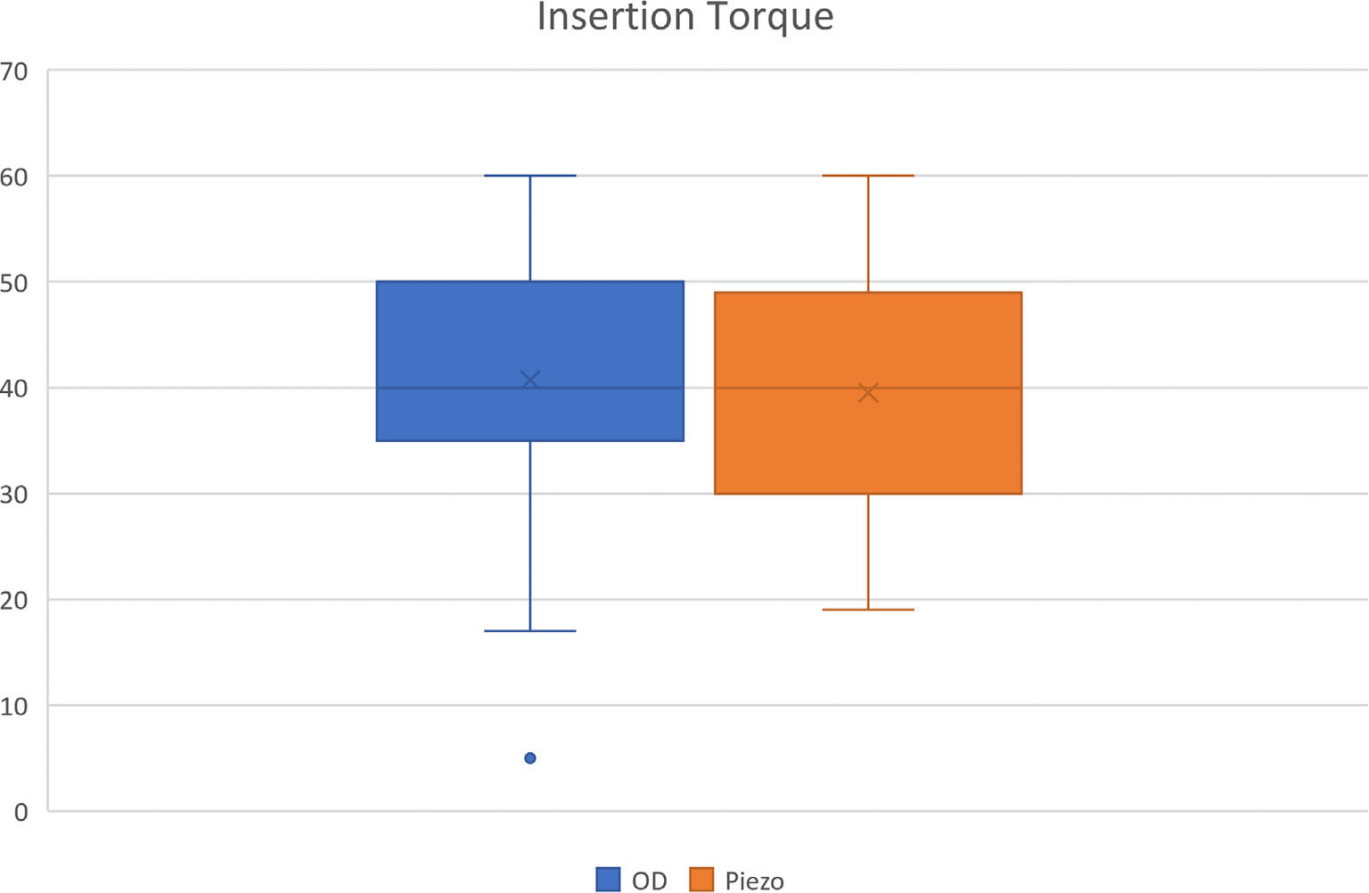
Two‐sample Wilcoxon rank‐sum test showed no significant differences in mean peak insertion torque between the two groups. Insertion torque is expressed in Ncm. OD: osseodensification drills (test); Piezo: piezoelectric tips (control)

Mean peak insertion torque was 40.7 ± 12.3 Ncm (range 5–60 Ncm) in the test group and 39.5 ± 10.2 Ncm (range 19–60 Ncm) in the control group. Two‐sample Wilcoxon rank‐sum test showed no significant differences (*p* = 0.47) between the two groups (Figure 3).

Mean ISQ values at baseline (primary stability) were 71.3 ± 6.9 and 69.3 ± 7.6 in the test and control group, respectively. Two‐sample Wilcoxon rank‐sum test showed no significant difference between the two groups (*p* = 0.48).

Implant stability decreased in both groups during the early healing period. The lowest peak was recorded 21 days after implant insertion for test group implants (mean ISQ 65.9 ± 7.8—7.6% decrease from mean primary stability) and 14 days after implant insertion for control group implants (mean ISQ 66.5 ± 6.7—4.9% decrease from mean primary stability).

**FIGURE 4:**
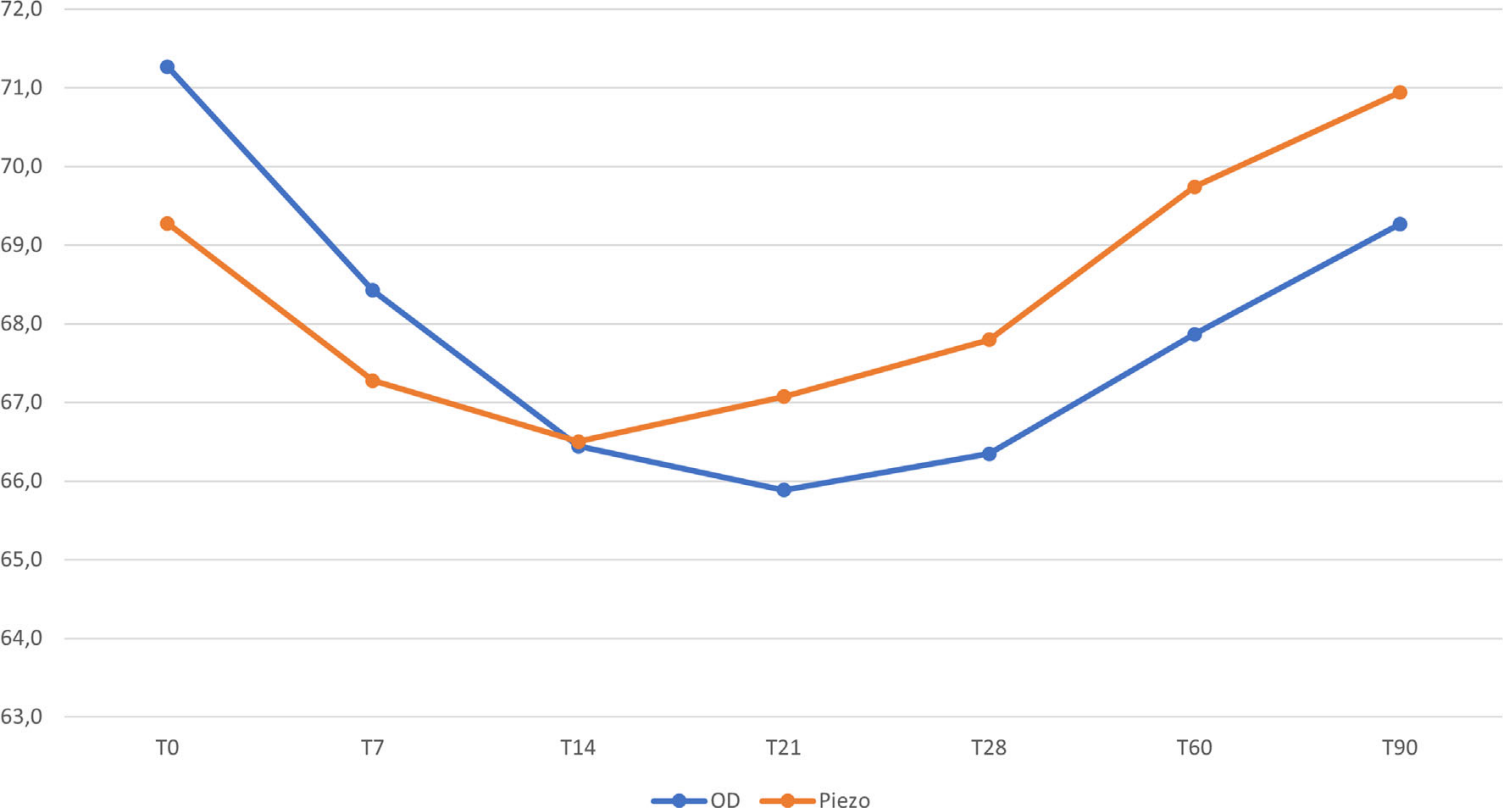
Changes in mean implant stability quotient values of both groups during the first 90 days after implant insertion. The lowest peak was registered at 14 days for the control group and at 21 days for the test group. OD: osseodensification drills (test); Piezo: piezoelectric tips (control)

After the third week, implant stability continued to increase constantly in both groups. However, only control group implants, at 60 and 90 days after implant insertion, showed ISQ values higher than at baseline (Figure 4).

Inter‐group comparisons were performed using the two‐sample Wilcoxon rank‐sum test and showed no significant differences in ISQ values between the test and control group during the entire period of observation (Table 2).

**TABLE 2:** ISQ values at the different time points in the test and control group

	OD	PISP	
Baseline	71.3 ± 6.9	69.3 ± 7.6	*p* = 0.48
7 days	68.4 ± 6.3	67.3 ± 6.1	*p* = 0.56
14 days	66.4 ± 8.5	66.5 ± 6.7	*p* = 0.81
21 days	65.9 ± 7.8	67.1 ± 5.7	*p* = 0.70
28 days	66.3 ± 6.8	67.8 ± 5.5	*p* = 0.50
60 days	67.9 ± 5.4	69.7 ± 5.4	*p* = 0.23
90 days	69.3 ± 5.4	70.9 ± 4.5	*p* = 0.30
	*p* = 0.06	*p* = 0.07	

*Note*: No inter‐ or intra‐group significant differences were demonstrated at any time point (*p* > 0.05). Data are expressed as mean ± standard deviation.

Abbreviations: OD, osseodensification drills; *p*, *p*‐value; PISP, piezoelectric implant site preparation.

Intra‐group comparisons, analyzed using the Scheffé test, showed no significant differences in ISQ values at any time point both in test and in control group (*p* = 0.06 and *p* = 0.07, respectively).

## 4 DISCUSSION

This multi‐center randomized clinical trial aims to compare primary and secondary stability of implants inserted into sites prepared with OD and piezoelectric tips. Being the very first analysis comparing OD and PISP, it was possible to examine and discuss only the available evidence on OD and PISP resulting from studies in which conventional drilling was used as comparator.

The present study was designed to control variables influencing implant primary stability, in order to highlight the impact of surgical technique on the healing process.^21^, ^22^ Each patient received two identical implants (same macrotopography, microtopography, diameter, and length) in sites with similar bone quality (adjacent or contralateral teeth in the upper premolar area) and implant site preparation was performed by experienced operators who underwent a calibration session prior to the study.

Primary stability was not significantly different between the test and control group either in terms of peak insertion torque (40.7 ± 12.3 Ncm in test group and 39.5 ± 10.2 Ncm in control group) or in ISQ (71.3 ± 6.9 and 69.3 ± 7.6 in test and control group, respectively). This outcome is indirectly confirmed by numerous studies on human subjects showing no significant difference in primary stability between implants inserted after conventional drilling when compared with PISP.^12^, ^23^, ^24^, ^25^, ^26^ Conversely, contrasting data are reported in clinical studies on OD for this topic. The majority of authors found no significant difference in implant primary stability between OD and conventional drilling,^27^, ^28^, ^29^ while one investigation highlighted the superiority of OD.^16^ This disagreement may be explained by inhomogeneity of implant design and differences in bone density among the cases of this latter study.^16^ Both of these factors can heavily influence implant primary stability.

In the context of the inflammatory response to surgical trauma, bone microdamage induces peri‐implant bone remodeling with definite steps (activation of osteoclast cutting cones, damaged bone removal by osteoclasts, pericytes recruitment and their differentiation into osteoblasts, and new bone formation by these osteoblasts).^30^ During the early phases of healing, osteoclastic activity reduces implant mechanical anchorage to the surrounding bone. Many studies have pointed out that, after conventional drilling preparation, implant stability tends to decrease significantly for the first 3 weeks after implant placement.^31^, ^32^, ^33^, ^34^, ^35^ In the present study, stability of implants inserted after PISP decreased for 14 days after placement (mean ISQ 66.5 ± 6.7–4.9% decrease from mean primary stability), in perfect accordance with previous clinical trials^12^, ^25^ and meta‐analyses.^36^, ^37^ Stability of implants inserted after OD decreased for 21 days after placement (mean ISQ 65.9 ± 7.8—7.6% decrease from mean primary stability), in accordance with a recent clinical study showing that the use of OD for implant site preparation does not prevent implant stability decrease during the first 3 weeks of healing.^38^ However, it should be underlined that ISQ value reductions recorded in the present study (both in test and control group) were very limited, without reaching statistical significance when compared to primary stability. In addition, inter‐group comparisons showed that also ISQ values measured in OD and PISP group resulted not significantly different during the entire period of observation. These results suggest that both techniques induce a very limited bone remodeling of the peri‐implant bone in comparison with conventional drilling preparation, in which significant loss of stability occurs during the first month after implant placement.^31^, ^32^, ^33^, ^34^, ^35^

After 12 months of prosthetic loading, 53 out of 54 implants were satisfactorily in function. One implant failed in the OD group before loading (1/27; 96.2% survival rate), while no failures were recorded in the PISP group (0/27; 100% survival rate). Also for this outcome, no significant differences were demonstrated between the two groups, in accordance with previous clinical studies and meta‐analyses reporting similar survival rates for implants inserted with different implant site preparation techniques.^16^, ^36^, ^39^, ^40^

It must be underlined that the findings of this multi‐center randomized clinical trial should be interpreted with caution due to some limitations of the present study. Factors including the limited numerosity of the sample, the selection of a specific surgical site (only lateral maxilla) and the use of a single implant type should be taken into consideration when generalizing the present results.

After analyzing data from the present study, it was not possible to reject the null hypothesis of this trial. In other words, no significant differences in implant stability were demonstrated between implants inserted into sites prepared with OD or piezoelectric surgery. Further, well‐designed preclinical studies and additional clinical trials are needed to better clarify the bone healing process after osseodensification procedures, and the possible benefits of this approach for dental implant therapy in low density bone.

## AUTHOR CONTRIBUTIONS

**Claudio Stacchi:** Concept/Design; data collection; data analysis/interpretation; drafting article; approval of article. **Giuseppe Troiano:** Statistics; data analysis/interpretation; drafting article; approval of article. **Graziano Montaruli:** Data collection; critical revision of article; approval of article. **Marco Mozzati:** Data collection; critical revision of article; approval of article. **Luca Lamazza:** Data collection; critical revision of article; approval of article. **Alessandro Antonelli:** Data collection; critical revision of article; approval of article. **Amerigo Giudice:** Data collection; critical revision of article; approval of article. **Teresa Lombardi:** Concept/Design; data collection; data analysis/interpretation; drafting article; approval of article.

## CONFLICT OF INTEREST

The authors declare no conflict of interest.

## Data Availability

The data that support the findings of this study are available from the corresponding author upon reasonable request.
